# Retraction: Eukaryote Culturomics of the Gut Reveals New Species

**DOI:** 10.1371/journal.pone.0346536

**Published:** 2026-04-06

**Authors:** The PLOS One Editors

The *PLOS One* Editors retract this article [1,2] due to concerns about compliance with the PLOS Human Subjects Research policy.

The Materials and Methods section in [1,2] reports sampling of stool specimens from individuals from Polynesia, Amazonia, Senegal, and India, but the article does not clarify when these samples were collected. The ethics approval reported in the article refers to agreement #09–022 issued by the Ethics Committee of the Institut Fédératif de Recherche 48 (IFR48), Marseilles, France. The article does not report prospective, local ethics approval from institutions or agencies in Polynesia, Amazonia, Senegal, or India. Furthermore, PLOS noted that the ethics approval number #09–022 was also reported in at least 247 other studies despite apparent differences in the aims and objectives, study locations, study populations, age ranges, methodologies, types of samples collected, and types of consent described in these studies. S1 File contains a summary of articles citing ethics approval number #09–022 of which PLOS is aware.

A representative from the Aix-Marseille Université stated that the institute disagrees with the retraction decision and that the study complied with applicable legislation and ethical standards. They stated that the samples collected in this study are considered to be human waste, and studies on human waste are not considered to be research involving the human person that would require ethics approval from a Comité de Protection des Personnes according to French law.

The Aix-Marseille Université representative provided the following ethics approval documentation for editorial review:

- Ethics approval document #09–022 issued by the IFR48 on December 9, 2009, for a study titled *Culture diversifiée des bactéties dans les selles humaines*.
- Ethics approval document #00081MSAS/DGS/DS/CNERS issued by the Ministère de la Santé et de l’Action sociale of Senegal on June 4, 2012, for a study titled *Identification des agents pathogènes responsables de fièvre au Sénégal. Réalisation de tests diagnostiques chez les maladies consultant dans les dispensaires de Dielmo, Ndiop, Niakhar, Mlopm, Bandafassi et Keur Momar Sarr.*
- Ethics approval documents #45/CEPF and #67/CEPF issued by the Comité D’Éthique de la Polynésie française on Dec 2, 2013, granting approval for a study titled *Epidémiologie et étude des microorganisms entériques isolés ou identifies à l’institut Louis Malardé.*

Furthermore, they clarified that the samples collected from two individuals from Amazonia were collected in a village in the Amazon rain forest, part of French Guiana, and state that the authorization from the ethics committee of French Polynesia also applies to the part of the study that took place in French Guiana since both are part of the French overseas territory (DOM-TOM).

However, editorial assessment of the responses and the documents by PLOS concluded that the documents and the institute’s response did not fully resolve the journal’s concerns. Specifically,

- The article does not report when the samples used in this study were collected. This information is needed to evaluate the article’s compliance with the PLOS Human Subjects Research policy.
- The document #09–022 provides approval for a study into bacteria, as opposed to eukaryotes, and lists the “Bacteriological laboratory of the Timone Hospital, Marseille, France” as the origin of the samples.
- Contrary to the institute’s statement that the samples from Amazonia were collected in a village in the Amazon rain forest, part of French Guiana, the fecal sample collection subsection of the article’s Materials and Methods states that these samples were collected from “two individuals from Amazonia (Manaus, urban area, forest area).” Manaus is located in Brazil, as opposed to French Guiana, and is not considered to be part of the French overseas territory. PLOS has not received any prospective local ethics approval documentation for the stool sample collected from the individuals in Manaus, Brazil.
• PLOS has not received any prospective local ethics approval documentation for the stool sample collected from the individual in New Delhi, India.

In addition, PLOS identified potential competing interests between the approving ethics committee that issued the document #09–022 and one or more of the article’s authors.

NG and MD did not agree with the retraction. DR either did not respond directly or could not be reached.

## Supporting information

S1 File List of 248 articles referencing ethics approval number N° 09–022. (XLSX)
